# Coupling Bacterioplankton Populations and Environment to Community Function in Coastal Temperate Waters

**DOI:** 10.3389/fmicb.2016.01533

**Published:** 2016-09-27

**Authors:** Sachia J. Traving, Mikkel Bentzon-Tilia, Helle Knudsen-Leerbeck, Mustafa Mantikci, Jørgen L. S. Hansen, Colin A. Stedmon, Helle Sørensen, Stiig Markager, Lasse Riemann

**Affiliations:** ^1^Centre for Ocean Life, Marine Biological Section, University of CopenhagenHelsingør, Denmark; ^2^Marine Biological Section, University of CopenhagenHelsingør, Denmark; ^3^Department of Bioscience, Aarhus UniversityRoskilde, Denmark; ^4^Centre for Ocean Life, National Institute of Aquatic Resources, Technical University of DenmarkCharlottenlund, Denmark; ^5^Laboratory for Applied Statistics, Department of Mathematical Sciences, University of CopenhagenCopenhagen, Denmark

**Keywords:** coastal ecosystems, community functions, bacterioplankton communities, extracellular enzymes, bacterial growth, 16S rRNA gene, seasonality, LASSO analysis

## Abstract

Bacterioplankton play a key role in marine waters facilitating processes important for carbon cycling. However, the influence of specific bacterial populations and environmental conditions on bacterioplankton community performance remains unclear. The aim of the present study was to identify drivers of bacterioplankton community functions, taking into account the variability in community composition and environmental conditions over seasons, in two contrasting coastal systems. A Least Absolute Shrinkage and Selection Operator (LASSO) analysis of the biological and chemical data obtained from surface waters over a full year indicated that specific bacterial populations were linked to measured functions. Namely, *Synechococcus* (*Cyanobacteria*) was strongly correlated with protease activity. Both function and community composition showed seasonal variation. However, the pattern of substrate utilization capacity could not be directly linked to the community dynamics. The overall importance of dissolved organic matter (DOM) parameters in the LASSO models indicate that bacterioplankton respond to the present substrate landscape, with a particular importance of nitrogenous DOM. The identification of common drivers of bacterioplankton community functions in two different systems indicates that the drivers may be of broader relevance in coastal temperate waters.

## Introduction

Coastal waters are among the most productive and biogeochemically active systems on Earth. Despite making up only about 7% of the global ocean surface, they sustain 14–30% of oceanic primary production (Gattuso et al., 1998). Coastal zones provide important ecosystem services in terms of biological productivity, assimilating terrestrial inputs, and biogeochemical cycling (Costanza et al., 2014). Factors influencing the productivity and stability of coastal ecosystems are therefore of particular interest. Heterotrophic bacterioplankton are one such factor, as they are the primary agents in degrading and assimilating dissolved organic matter (DOM). Estuarine systems receive terrestrial DOM which together with the autochthonous production support bacterial growth and activity (Hopkinson and Smith, 2005). The autochthonous DOM load is high in some estuaries where elevated inorganic nutrient concentrations fuel a high primary production (Markager et al., 2011).

In the bacteria-DOM interface, the production of extracellular enzymes and subsequent uptake of hydrolyzed compounds is the rate-limiting step of bacterial DOM utilization (Arnosti, 2011). Enzymatic degradation of polymers is necessary because bacterial uptake is limited to molecules <600 Da (Weiss et al., 1991). Since enzyme profiles and activity as well as substrate uptake differ between groups of bacteria (Martinez et al., 1996; Alonso-Sáez and Gasol, 2007), a linkage between community composition and the utilization of DOM (i.e., community function) may be hypothesized. However, while such linkage has been demonstrated in some cases (e.g., Langenheder et al., 2006; Teske et al., 2011; Logue et al., 2015), others find that community functions may remain relatively stable despite large underlying changes in community composition (Fernández et al., 1999; Comte and Del Giorgio, 2009; Sjöstedt et al., 2013). A complicating factor is the intricate and continuous interactions between bacterial population dynamics, their function, and environmental conditions. Bacterial community composition is reckoned to respond to environmental conditions (Gasol et al., 2002), which may also have an effect on community functions (Findlay et al., 2003; Kirchman et al., 2004; Judd et al., 2006). Community composition has been linked to community functions of varying specificity, e.g., extracellular enzyme rates or preference for different DOM fractions (Langenheder et al., 2006; Alonso-Sáez and Gasol, 2007; Teira et al., 2008; Logue et al., 2015). However, the distribution of bacterial functions in a community may vary with the phylogenetic level (Martiny et al., 2013) challenging attempts to assign taxonomic information to particular bacterial community functions. Furthermore, some functions may be so fundamental in character and widely distributed (e.g., biomass and respiration) that they appear independent of community composition dynamics (Langenheder et al., 2005). Hence, it remains unclear how bacterial population dynamics influence community function and what implications they have for coastal biogeochemical cycling.

Recent studies have shown the potential of high resolution data and sampling (Lindh et al., 2015; Logue et al., 2015), and the value of discriminating between compositions of total (16S rDNA) and active (16S rRNA) communities (Jones and Lennon, 2010; Franklin et al., 2013) in investigations of the ecological role of bacterioplankton community composition. Indeed, the observed disparity between rRNA and rDNA communities (Campbell et al., 2011; Franklin et al., 2013) raises concerns regarding the common and exclusive use of the 16S rRNA gene, for characterization of communities when attempting to identify drivers of community function. The use of rRNA must be applied with caution, as some taxa demonstrate highly unstable rRNA – growth relationships (Blazewicz et al., 2013). The application of rRNA may, nonetheless, prove useful, as non-growth functions (e.g., excretion of extracellular enzymes and uptake) are important in regulating community functions and may display a decoupling from growth (Karner and Herndl, 1992). High taxonomic resolution may also be important when inferring links to function. A recent study demonstrates how the abundances of dominant bacterial classes were directed by the dynamics of only a fraction of the underlying OTUs (Ruiz-González et al., 2015b). This suggests that single OTUs at times drive community functions, and such links between taxa and function may not be discernible if community analyses are carried out at the class or phylum level.

In temperate regions, seasonality exerts strong control over bacterial abundance and activity and induces large changes in community composition (Alonso-Sáez et al., 2007; Lindh et al., 2015) and function (Karner and Rassoulzadegan, 1995; Sherry et al., 1999; Lemée et al., 2002; Reinthaler and Herndl, 2005). Long-term studies have documented highly repeatable patterns of seasonality in composition (Fuhrman et al., 2006; Gilbert et al., 2012). Consequently, systems characterized by strong seasonality offer natural scenarios well-suited for studying interactions between bacterioplankton community composition, functionality and the environment.

The aim of the present study was to identify links between total or active bacterial populations and community function, and how seasonal dynamics influence bacterioplankton communities. By examining two sites of contrasting nutrient richness over a year, we sought to infer links between community composition and function in Danish coastal waters.

## Materials and Methods

### Sites, Sampling and Environmental Parameters

Roskilde Fjord (RF) is a shallow (average depth of 3 m), enclosed estuary with a mean water residence time of ∼1 year and high nutrient load from urbanization and agriculture within the catchment (Pedersen et al., 2014). The station (55°42.00′N, 12°04.46′E) is located in the southern basin with a depth of 5 m. In January, RF was inaccessible due to ice cover; therefore, water was collected from a pier (55°41.49N, 12°04.93′E) 1 km southeast of the station. The station in Great Belt (GB) is located in the middle of the strait with a depth of 35 m (55°30.27′N, 10°51.43′E). The belt has a mean depth of 17 m and is characterized by strong currents and stratification caused by the outflow of brackish water from the Baltic Sea at the surface and inflow of saline seawater from the North Sea at the bottom. Unfiltered seawater was collected from surface waters (1 m), using 5 l Niskin bottles, every month from RF and GB during 2012. Samples were always obtained at 10:00 – 13:00 h and all rate measurements and fixations carried out within 1–3 h after sampling. All incubations (e.g., extracellular enzyme assay, Biolog plates, bacterial production (BP), and respiration) were kept in the dark at temperatures as close as possible to the *in situ* temperature with a maximum difference of ±3.2°C. Data on Chlorophyll *a* (Chl *a*), dissolved organic carbon (DOC), inorganic nutrients, temperature, and salinity are published in Bentzon-Tilia et al. (2015).

Colored dissolved organic matter (CDOM) samples were collected using pre-combusted Whatman^®^ GF/F filters (Sigma–Aldrich, St. Louis, MO, USA). Absorption and fluorescence characteristics were measured on a Horiba Scientific Aqualog, processed according to Murphy et al. (2011). CDOM absorption properties were characterized by calculating the spectral slope across 275–295 nm, 300–450 nm (Helms et al., 2008), and 300–650 nm (Stedmon et al., 2000). Fluorescence excitation-emission matrices were characterized using parallel factor analysis (PARAFAC) with the drEEM toolbox (Murphy et al., 2013) as well as reporting the fluorescence intensities for the commonly reported wavelength regions: A, M, C, and T (Coble, 2007). Bioavailable DOC was estimated through degradation experiments and calculated using an exponential regression model: DOC(t) = RDOC + BDOC exp^(-D_t_ .t)^, where RDOC is the refractory pool of DOC, D_t_ is a time coefficient for degradation of DOC and t is the time in days (Hansen and Bendtsen, 2014).

### Extraction of Nucleic Acids and 16S rRNA Amplicons Sequencing

Extractions of DNA and RNA, and cDNA synthesis were done as described in Bentzon-Tilia et al. (2015). 16S ribosomal RNA (cDNA) and DNA amplicons of the V4 region of bacterial and archaeal communities were obtained using the forward and reverse primers 515f (5′-GTGCCAGCMGCCGCGGTAA) and 806r (5′-GGACTACHVGGGTWTCTAAT), respectively (Caporaso et al., 2012). Polymerase chain reactions (PCRs) were performed in 20 μl reaction volume containing 10 ng template DNA or cDNA, primers and PureTaq Ready-to-go PCR beads (GE Healthcare, UK). The PCR conditions included an initial denaturing step at 94°C for 3 min followed by 29 cycles of 94°C for 45 s, 50°C for 1 min, 72°C for 1 min 30 s, and a final step of elongation at 72°C for 10 min. Triplicate PCR reactions were pooled for each sample, purified using the Agencourt AMPure XP purification kit (Beckman Coulter Inc., Brea, CA, USA), and quantified using the Quant-iT^TM^ PicoGreen^®^ quantification kit (Invitrogen, Waltham, MA, USA) and a FLUOstar OPTIMA plate reader (BMG Labtech GmbH, Ortenberg, Germany). PCR amplicons were pooled in equimolar concentrations and subjected to paired-end sequencing on an Illumina MiSeq (National High-throughput DNA sequencing Centre, University of Copenhagen, Denmark).

Sequence reads were assembled, trimmed to a mean length of 252 nucleotides, and de-multiplexed using QIIME v1.9 (Caporaso et al., 2010). Removal of singletons and clustering of operational taxonomic units (OTUs) at 97% similarity was done in USEARCH v1.8 (Edgar, 2010) using the UPARSE-OTU algorithm (Edgar, 2013) with implicit chimera check. Taxonomy was assigned in QIIME using uclust (Edgar, 2010) and the Greengenes v.13.8 reference database (Mcdonald et al., 2012). OTUs failing taxonomy assignment were manually checked using the basic local alignment search tool BLASTN (Morgulis et al., 2008), through the NCBI online access repository^1^, removing any chimeras overlooked during the implicit chimera check. Chloroplasts and mitochondrial reads were removed before downstream analysis. OTUs only occurring once in the dataset and/or included <10 reads in total were excluded. For visual representations of relative abundances and analyses on community compositions, samples were rarefied to a sample size of 16,000 reads per sample (smallest sample size 16,016) using the QIIME v1.9 implementation of the Mersenne twister pseudorandom number generator. Patterns in community composition were visualized using the Bray–Curtis dissimilarities and hierarchical clustering using the function hclust() in R. Henceforth, 16S rRNA and rRNA gene amplicons are referred to as rRNA and rDNA, respectively. Sequences were deposited to GenBank NCBI (accession numbers KU674968-KU677976).

### Community Functions

Community functions were assessed using bulk water, unless otherwise stated. The potential capacity for substrate utilization by the microbial communities was assessed using EcoPlates^TM^ (Biolog Inc., Hayward, CA, USA), inoculated with 125 μl sample and immediately read on a FLUOstar OPTIMA plate reader (BMG, Labtech GmbH, Ortenberg, Germany). Plates were read with 1–2 days intervals until maximum average well color development (AWCD) was reached, usually within 2 weeks. The AWCD ((∑ (R-C))/93) was calculated as the sum of color development in the plate, expressed as an average per well, based on the blank (C) corrected absorbance value (R) of each well (Garland et al., 2001). The time point where the AWCD was closest to 0.5 was chosen to calculate the mean color development of each blank-corrected well. The absorbance values were then processed and normalized using the AWCD value as described in Comte and Del Giorgio (2009). For the absence-presence summary of substrates being used over the year, a substrate was scored as positive if minimum one replicate well reached an absorbance value of ≥1 in the normalized dataset. No data are available for the EcoPlates^TM^ from January, February, May, and June in the RF and February, May, and June in the GB samples.

Extracellular enzyme activities were assayed using fluorogenic 4-methylumbelliferone (MUF) and 7-amino-4-methylcoumarin (MCA) substrates (Sigma–Aldrich, St. Louis, MO, USA). The enzyme assays were prepared according to Hoppe (1983) modified to a reaction volume of 200 μl with 400 μM substrate (final concentration). The enzymes assayed and substrates used were: protease (L-Leucine-MCA), chitobiase (MUF-*N*-acetyl-β-D-glucosaminide), lipase (MUF-oleate), and α- and β-glucosidases (MUF-α-D-glycopyranoside and MUF-β-D-glycopyranoside, respectively). Assays were measured in five replicates at 355 nm excitation and 460 nm emission on a FLUOstar OPTIMA plate reader (BMG, Labtech GmbH, Ortenberg, Germany). No data are available for the enzymes from January and June in RF and February and June in GB.

Bacterial production and abundance was followed in both bulk and GF/C filtered water, using ^3^H-thymidine incorporation (Fuhrman and Azam, 1982) and flow cytometry, respectively. A conversion factor of 1.4 × 10^18^ cells mol^-1^ thymidine was used (Fuhrman and Azam, 1982). Detailed methods and values for bulk water are published in Bentzon-Tilia et al. (2015). Bacterial Growth Efficiency (BGE) was calculated using:

(1) $$BGE\;=\;\frac{BP}{BP\;+\;BR}\;100$$

Measurements of bacterial respiration (BR) and production, specifically used for the calculation of BGE, were obtained from water filtered through pre-combusted Whatman^®^ GF/C filters (Sigma–Aldrich, St. Louis, MO, USA), to remove larger organisms. Respiration was measured as oxygen consumption in four to five replicate 100 ml BOD bottles incubated in water baths and measured every 24 h for 3 days, using the FireSting contactless sensor system (PyroScience GmbH, Aachen, Germany). Respiration rates were estimated by linear regression assuming a respiratory quotient of 1. BGE data are not available from January, February, and June in RF and February, May, and June in GB.

### Data Analyses

All data and statistical analyses were carried out in R (R Development Core Team, 2015)^2^ using the R packages glmnet v. 2.0-2 (Friedman et al., 2010), and vegan v. 3.2.2 (Husson et al., 2015; Oksanen et al., 2015). The Least Absolute Shrinkage and Selection Operator (LASSO) analysis was applied to identify potential drivers of bacterial community composition, due to the data structure with many more parameters than measurements (*p* >> *n*), which if left unaddressed, will report spurious correlations. The analysis builds on the “bet of sparsity” principle (Hastie et al., 2009) assuming that only few of the coefficients are non-zero, and thus, reducing the risk of spurious correlations.

Patterns of potential substrate utilization by the communities were based on 31 different substrates (Ecoplates) and scored as positive if at least one out of three replicates had an absorbance value higher than the AWCD, which after normalization equates to 1. The potential substrate use (Euclidean distances of normalized data) were analyzed against the community composition of the total and active bacterial communities (Bray–Curtis dissimilarities of both rarefied and non-rarefied communities) using a Mantel’s test (9999 permutations, Pearson’s correlations). A permutational analysis of variance (PERMANOVA) using the adonis() function in vegan and Bray–Curtis dissimilarity matrices was performed to examine the utilization patterns over the year.

The LASSO analysis was used to evaluate eight different community functions (response) against the proportional abundance (%) of total and active OTUs (3,009) in the communities, and 35 different environmental parameters to identify predictor variables (a list of the community functions and environmental parameters is given in Supplementary Table S1). For each selected predictor variable a Pearson correlation with the community function was computed and denoted *r*. These analyses were carried out separately for RF and GB, and the final model variables were then selected against the criteria of: (i) co-occurrence in systems (i.e., the predictor variable is selected for both RF and GB) and (ii) the two correlation coefficients of a variable should have the same sign. The use of co-occurrences between the two different systems increased the validity of the identified couplings. Only LASSO models containing one or more variables passing the criteria were further processed. The site-specific *r*-values for the selected variables along with a common *R*^2^-value across sites and a common Root Mean Squared Prediction Error calculated as: RMSP = 
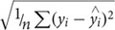
, are reported in Supplementary Table S2. Note that RMSPE maintains the unit of the function variable.

## Results

### Environmental Parameters

**FIGURE 1:**
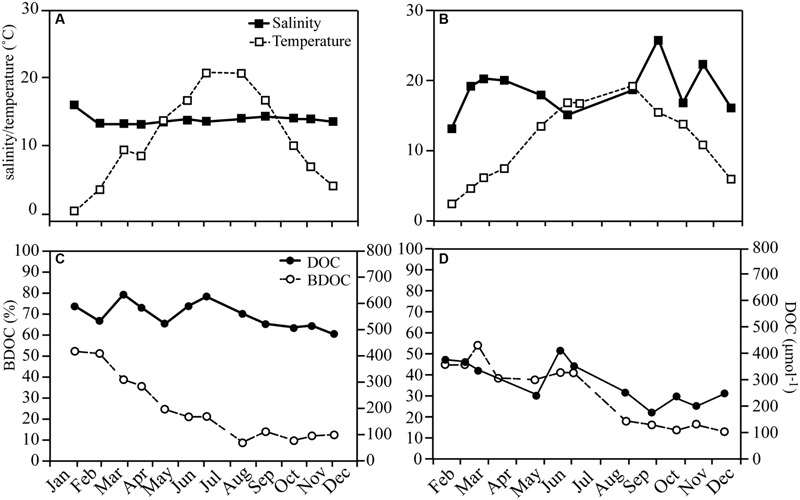
**Seasonal variations in Roskilde Fjord (Left side panel) and Great Belt (Right side panel) of salinity and temperature **(A)** and **(B)** and in DOC and bioavailable DOC (BDOC) as % of total DOC **(C)** and (D)**.

Salinity and water temperature varied between 12–15 and 0.6–20°C in RF, and 11–24 and 0.4–17°C in GB (**Figures 1A,B**). DOC concentrations varied between 511–714 and 184–418 μmol C l^-1^ in RF and GB, respectively. Bioavailable DOC roughly followed total DOC concentrations over the year in both systems, showing a decreasing trend from the beginning of the year, and accounting for 9–52 and 6–47% of total DOC for RF and GB, respectively (**Figures 1C,D**). The PARAFAC model identified four fluorescent DOM components C1-4, in both systems (Supplementary Figure S1). C1-3 had broad fluorescence maxima at visible wavelengths, while C4 had a UVA fluorescence peak. A comparison of the spectral properties of the components with previous studies was done using the OpenFluor database (Murphy et al., 2014). Matches were found for all four components linking C1 and 3 to terrestrial humic-like components, and C2 and C4 to a microbial humic-like and protein-like component, respectively (Supplementary Figure S1). The relative contribution of each fluorescent component and the CDOM absorption spectral slope values indicate qualitative seasonal changes in DOM in connection with the spring bloom and mixing (salinity), with a more pronounced effect in GB than RF (Supplementary Figure S2).

**FIGURE 2:**
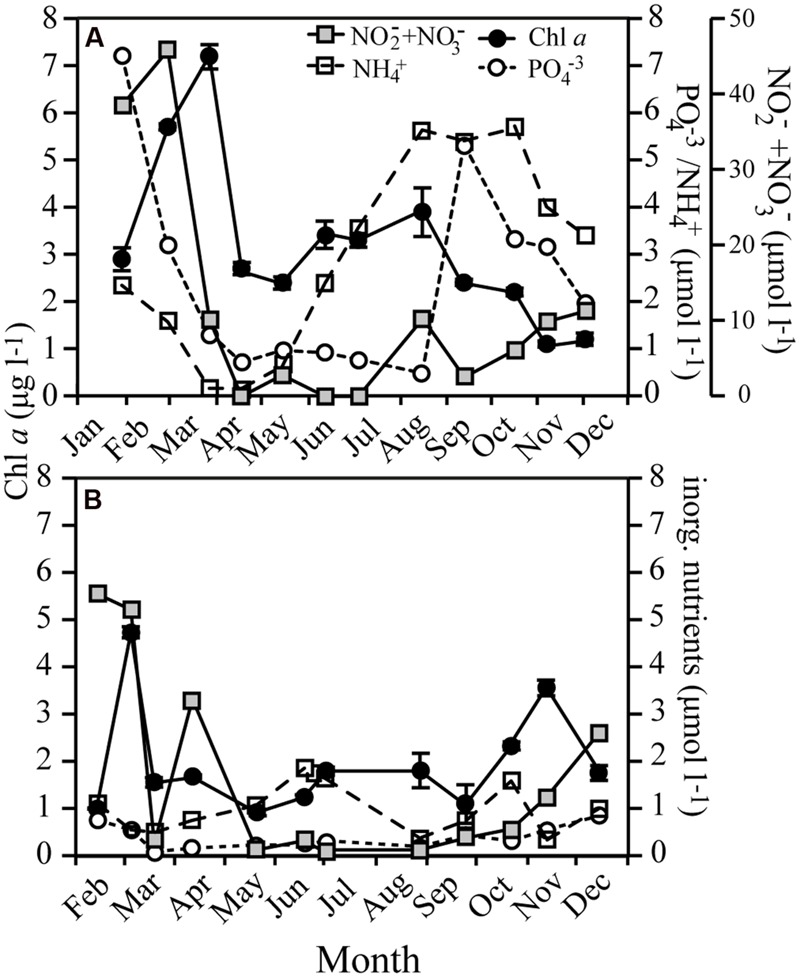
**Seasonal variations in Chl *a* and the concentration of inorganic nutrients for Roskilde Fjord **(A)** and Great Belt **(B)**.** Note the separate *y*-axes for the inorganic nutrients in **(A)**. Error bars represent standard error.

Several phytoplankton blooms occurred over the year in both systems (**Figure 2**), with the spring bloom from February to March and reaching maximum Chl *a* concentrations of 7.2 and 4.7 μg l^-1^ in RF and GB, respectively. In RF, Chl *a* increased again over the summer peaking in August (3.9 μg l^-1^), while GB experienced a bloom in October–November (3.6 μg l^-1^). The spring blooms caused large decreases in concentrations of inorganic nutrients in both systems, in particular inorganic nitrogen (**Figure 2**).

### Bacterial Growth

**FIGURE 3:**
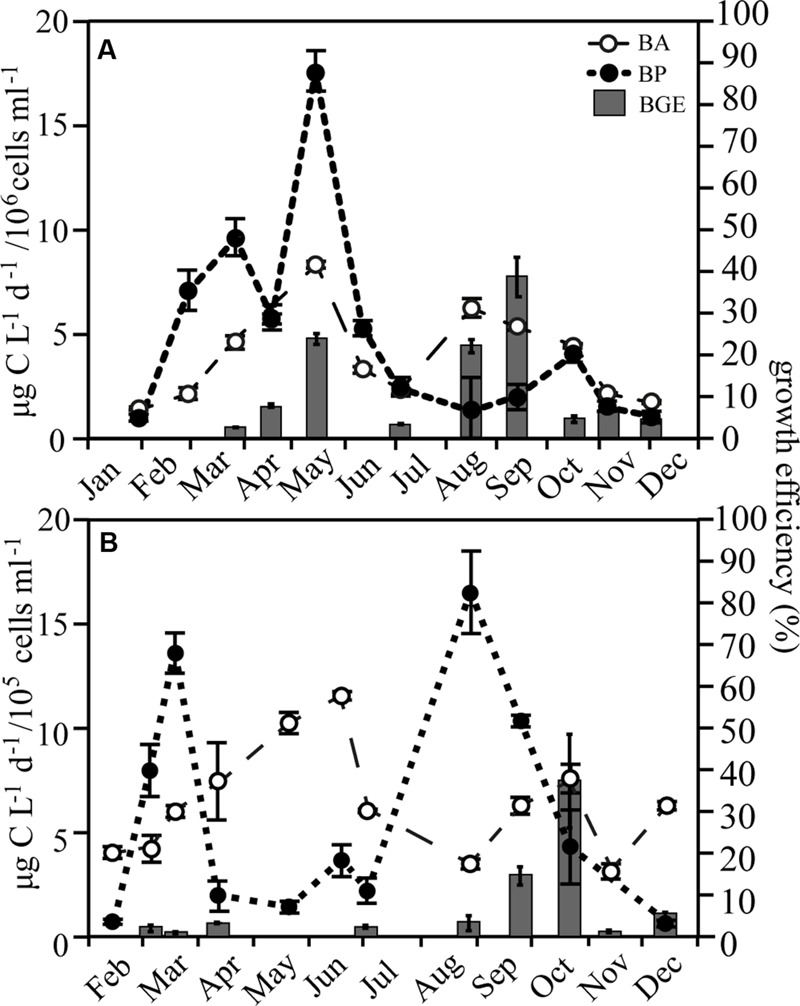
**Bacterial abundance (BA), production (BP), and growth efficiency (BGE) in Roskilde Fjord **(A)** and Great Belt **(B)**, respectively, over the season.** Note the different scales for BA in **(A)** and **(B)**. Error bars represent standard errors.

Bacterial abundance in RF was on average 4.0 ± 2.1 × 10^6^ cells ml^-1^, while being roughly tenfold lower in GB (6.4 ± 2.7 × 10^5^ cells ml^-1^; **Figure 3**; Supplementary Figure S3). BP in RF increased with Chl *a* during the spring bloom, followed by a second, larger peak in May, which coincided with maximum bacterial abundance. BGE increased on two occasions in RF with maxima in May (24 ± 2.9%) and September (39 ± 9.4%; **Figure 3A**). In GB, the production peaked concurrently with the spring bloom and again in August. However, in GB the growth efficiency was <4% during spring and early summer despite high BP, while in the fall the growth efficiency increased to a maximum value of 37 ± 11% following the largest peak in production (**Figure 3B**).

### Extracellular Enzymes and Carbon Utilization

**FIGURE 4:**
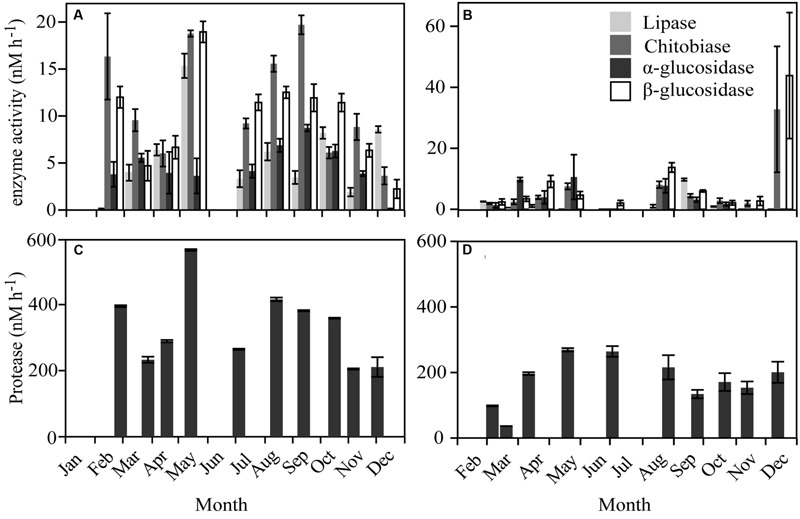
**Enzymatic activities over the year in Roskilde Fjord (Left panel) and Great Belt (Right panel).** The activities for lipase, chitobiase, α- and β-glucosidase in **(A)** and **(B)**, note differences of *y*-axes. Protease activities are shown separately in **(C)** and **(D)** due to higher rates.

**FIGURE 5:**
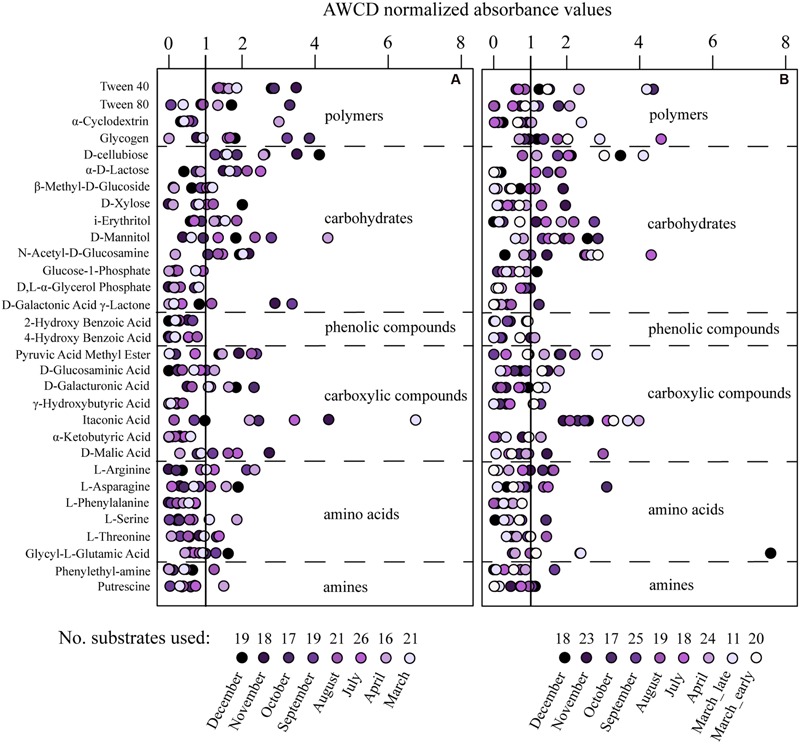
**The capacity for substrate utilization using Biolog Ecoplates in the communities from Roskilde Fjord **(A)** and Great Belt **(B)**.** The absorbance values were normalized to the average well color development (AWCD) of the plate, and months scoring higher than the average well color (value of 1) is interpreted as the substrate being utilized. Each point represent the average of triplicate wells.

Extracellular enzyme activities varied over the year with average rates higher in RF than GB (**Figure 4**; Supplementary Figure S4). In both systems protease activities were on average 100-fold higher than the other enzymes (**Figure 4**). Most of the 31 different substrates were utilized on one or several occasions and varied over the year in both RF and GB (**Figure 5**). In RF, the utilization of six substrates (Glucose-phosphate, Glycerol phosphate, Hydroxybutyric acid, Phenylalanine, 2- and 4-Hydroxybenzoic Acid.) was never detected, while Tween 40 and Cellobiose were utilized on all occasions. In GB, utilization of three substrates (Glycerol phosphate, Phenylalanine and 2-Hydroxybenzoic acid) were not detected, while Itaconic acid were utilized on all occasions. Overall patterns in substrate use correlated to months (PERMANOVA, permutations = 999) with the months alone explaining *R*^2^ = 0.407 (*p* = 0.001) and *R*^2^ = 0.586 (*p* = 0.001) of the variance in RF and GB, respectively. However, the patterns of potential substrate utilization over the year were not significantly linked to the community composition of the total or active communities (Mantel test, 9999 permutations, *p* > 0.1 for all combinations tested).

### Community Composition

**FIGURE 6:**
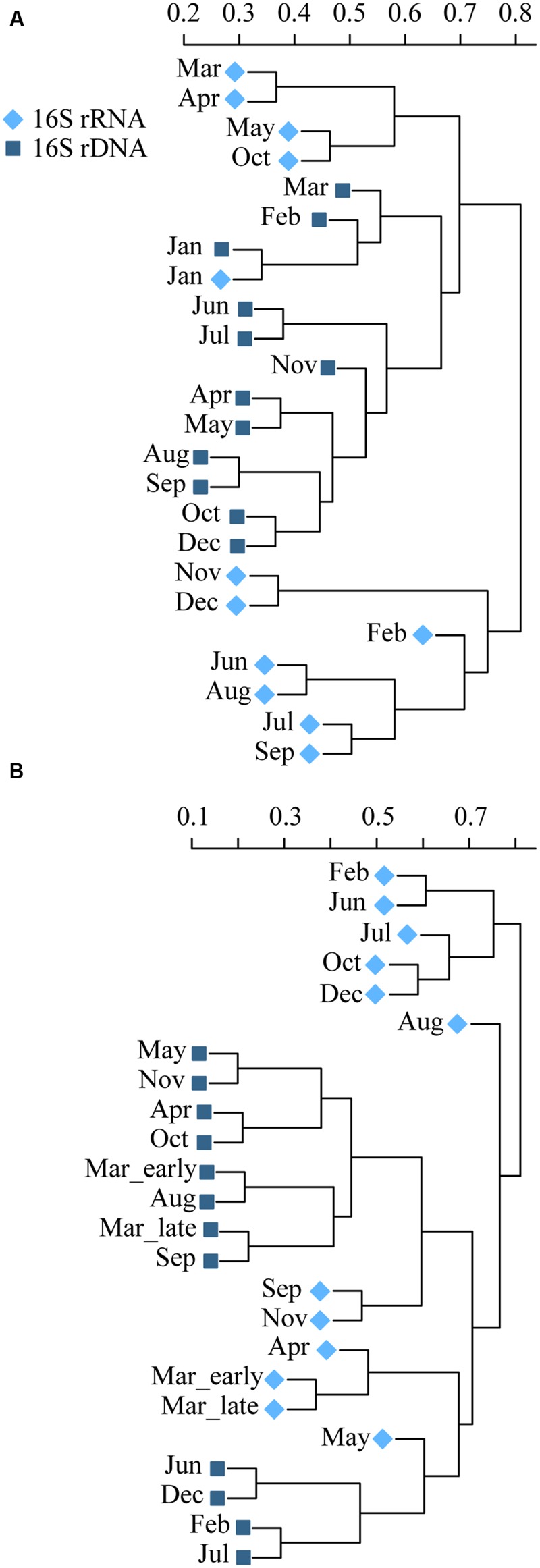
**Dendrogram using Bray–Curtis dissimilarites and hierarchical clustering, showing differences in community compositions between total (rDNA, ■) and active (rRNA, ◆) Bacteria and Archaea over the year in Roskilde Fjord **(A)** and Great Belt (B)**.

A total of 1,467,365 high quality reads remained after quality and chimera check, with 12,057 reads assigned to *Archaea*. Clustering at 97% similarity resulted in a total of 3,009 OTUs. Patterns in community composition differed over the year between the total (rDNA) and the active bacteria (rRNA; **Figure 6**; Supplementary Figure S5). In RF, the total communities formed a distinct group of sub clusters of neighboring months indicating seasonal progression, which to some degree was mirrored by the active communities (**Figure 6A**). In January exclusively, the total and active communities were more similar than to any neighboring month’s community. In GB, the total and active communities formed several discrete clusters, with no clear seasonal progression (**Figure 6B**). The phyla *Proteobacteria, Bacteroidetes, Actinobacteria*, and *Cyanobacteria* dominated over the year in both the total and active communities in RF and GB (Supplementary Figure S5). They made up between 82 – 93 and 80 – 94% of the total communities in RF and GB, respectively. Similarly, they made up between 85 – 98 and 81 – 97% in the active communities in RF and GB, respectively.

### Potential Drivers of Community Functions

**FIGURE 7:**
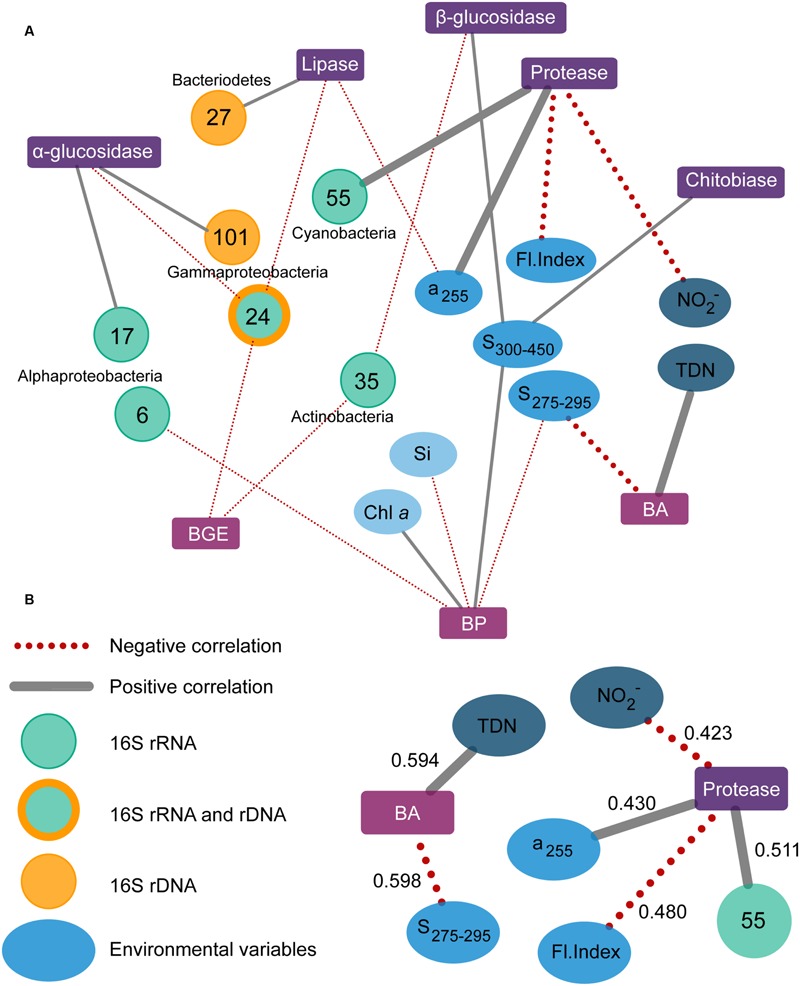
**Association network over all correlations between community functions (squares) and variables of bacterial populations (OTUs, circles) or environmental parameters (ellipses).** Community functions include the activity of five different extracellular enzymes, and bacterial growth efficiency (BGE), bacterial production (BP), and abundance (BA). Positive and negative correlations are shown as gray and red dotted lines, respectively, with thick lines indicating correlations with *R*^2^ > 0.4 **(A)**. A cutoff of 0.4 was used for determining strong correlations, which are shown separately **(B)** with their *R*^2^-values. TDN, total dissolved nitrogen; Si, dissolved silicate; Fl; Index, fluorescence index.

The LASSO analyses were used to identify and evaluate links between bacterial community functions, total and active OTUs, and environmental parameters. The community functions, for which the analyses produced variables, are depicted in **Figure 7A**, and grouped according to function type, i.e., extracellular enzymes and bacterial growth, with links to bacterial populations (OTUs) or environmental parameters. The LASSO model outputs identified specific OTUs from the major taxonomic groups of *Gamma-* and *Alphaproteobacteria, Bacteroidetes, Actinobacteria*, and *Cyanobacteria* (see **Table 1**). The most important environmental variables identified by the LASSO models were all related to aspects of DOM and nitrogen availability, and to a lesser extent phytoplankton, inorganic nutrients, and temperature. Variables were sorted by their *R*^2^-value, using 0.4 as a cut-off for defining strong correlations. The full list of analyzed community functions is provided in Supplementary Table S1 and the output in Supplementary Table S2 reporting *r*-, *R*^2^-, and RMSPE-values. The patterns of potential substrate utilization suggested a seasonal component, as the months correlated with these patterns. Overall, the analyses suggested that specific bacterial populations, specific environmental conditions, and the season were potentially important for the function of the bacterial community.

**Table 1:** Operational taxonomic units (OTUs) appearing in one or more LASSO model.

OTU#	Community	Phyla/class	Taxa
OTU 55	rRNA	*Cyanobacteria*	*Synechococcus*
OTU 27	rDNA	*Bacteroidetes*	*Flavobacterium*
OTU 35	rRNA	*Actinobacteria*	*Actinomycetales*
OTU 17	rRNA	*Alphaproteobacteria*	*Pelagibacteraceae*
OTU 6	rRNA	*Alphaproteobacteria*	*Caulobacteraceae*
OTU 24	rRNA/rDNA	*Gammaproteobacteria*	*Pseudoalteromonas*
OTU 101	rDNA	*Gammaproteobacteria*	*Vibrio*

*Phylogeny at 97% similarity of the 16S rRNA gene (rDNA) and rRNA using the Greengenes database v. 13.8 with the highest taxonomic resolution listed in Taxa. rRNA/rDNA = the OTU appeared in one or more LASSO model with both rDNA and/or rRNA. The phylum *Proteobacteria* is resolved to class.*

## Discussion

The aim of this study was to identify general drivers of bacterioplankton community functions, taking into account the dynamic variability of bacterial populations and environmental conditions over seasons, in two coastal systems. The LASSO models indicated that specific populations within *Cyanobacteria, Bacteroidetes, Actinobacteria, Alpha-*, and *Gammaproteobacteria* were associated with extracellular enzyme activity and growth efficiency. However, most of the measured functions had weak or no correlation to community components. The observed dynamics of both community composition and function appeared to be governed by nitrogen availability and DOM.

### The RF and the GB: Two Contrasting Systems

The two examined systems differed in basic parameters, e.g., inorganic nutrients, DOM and phytoplankton biomass, reflecting trophic status, with RF being the most eutrophic system. Bacterial community functions also varied between the systems and over the year, with RF in general supporting higher cell abundances and extracellular enzyme rates than GB. The growth efficiency patterns showed that bacteria in both systems allocate a high proportion of processed carbon into growth during late summer and fall. In RF, a period of high growth efficiency also occurred during the spring bloom. In contrast, the growth efficiency was low in GB during spring. Bacterial community compositions differed between RF and GB, and only RF showed a seasonal progression by the clustering of months. In general, the active communities at both sites were more similar to each other than when comparing the total communities, with the exception of RF in January. This difference between active and total communities could be due to large proportions of the community being capable of switching to dormancy (Jones and Lennon, 2010). Temperate systems like RF and GB exhibit significant seasonal changes and dormancy is a likely strategy to survive unfavorable conditions, which may preserve populations by cycling them in and out of a seed bank (Campbell et al., 2011). Populations cycling between dormant and active states could be important in explaining the dynamics of community functions over seasons.

### Environment and Bacterial Populations as Drivers of Bacterioplankton Functionality

By selecting only variables for which correlations occurred in both systems the importance of a variable was substantiated by independent data sets, thus reducing the risk of picking random variables. It must be noted, though, that the presented analyses do not consider time-lagged relationships, which have previously been observed for microbe-microbe and microbe-environment interactions in waters at the San Pedro Ocean Time-series location (Fuhrman and Steele, 2008; Needham and Fuhrman, 2016). For a number of community functions correlations with variables were relatively strong, with values of *R*^2^ > 0.4. These were bacterial abundance and protease activity (see **Figure 7B**). Bacterial abundance correlated positively with total dissolved nitrogen presumably driven by dissolved organic nitrogen, as inorganic nitrogen was measured directly and did not correlate with abundance. The relationship likely reflects the role of bacteria in processing dissolved organic nitrogen from phytoplankton. Additionally, abundances correlated negatively with the slope S_275-295_ – a proxy of CDOM molecular weight, which is inversely related to S_275-295_ (Helms et al., 2008). Slopes of CDOM generally have low values, which increase with, for example, exposure to photo-degradation. The negative relationship observed at both sites could reflect changing quality of DOM present and the subsequent response in bacterial abundances. The coupling of bacterial abundance to total dissolved nitrogen and S_275-295_ indicates a link between bacterial abundances and the supply and character of DOM.

Proteases play an important role in microbial nitrogen acquisition by hydrolyzing nitrogen-rich substrates such as proteins, and their activity is indicative of nitrogen deficiency (Sala et al., 2001; Church, 2008). Protease activity correlated positively with a cyanobacterium *Synechococcus* (OTU_55), which reached abundances of 8 and 6% in the RF and GB active communities, respectively (Supplementary Figure S6). Several marine *Synechococcus* are capable of producing extracellular proteases, and *in situ* protease activities have been correlated to the abundance of *Synechococcus* in marine waters (Martinez and Azam, 1993). Both RF and GB experience periods with low levels of inorganic nitrogen where proteases likely would support the microbial nitrogen demand, consistent with the negative correlation between protease activity and nitrite (**Figure 7B**). Furthermore, protease activity correlated negatively with the Fl. Index, which distinguishes microbial (>1.7) from terrestrially (∼1.4) derived fulvic acids (Mcknight et al., 2001). The Fl. Index signals in both RF and GB indicate that most fulvic acids are autochthonous, and phytoplankton is a known major source of marine fulvic acids (Mcknight et al., 1991). Protease activity correlated positively with UV-absorption (255 nm), which indicate DOM quantity (Stedmon and Nelson, 2014). Comparing the average S_275-295_ values, 12.0 and 15.7 for RF and GB, respectively, there are evident differences between sites in the degree of degradation of the terrestrial DOM. GB represents aged organic material exported from surface waters of the Baltic Sea, which has been exposed to photo-degradation (Osburn and Stedmon, 2011). The LASSO model variables suggest the load and quality of DOM and periodically active bacterial populations, in this case a *Synechococcus*, may contribute to drive overall protease activity. Together, the models for bacterial abundance and protease activity imply an occasional microbial nitrogen limitation in both systems.

For most community functions correlations with variables were relatively weak, with values of *R*^2^ < 0.4. This may in part be due to a high redundancy of certain functions in natural bacterioplankton communities, consistent with previous observations of functional equivalence between distinct communities (Langenheder et al., 2006). All active populations likely contribute to core community functions, such as growth and respiration, through expression of basic physiological traits. Such core functions may therefore be naturally resistant toward analyses attempting to identify relationships to taxonomically defined populations. Furthermore, variability in the phylogenetic conservation of bacterial traits (Martiny et al., 2013) suggests that relationships between composition and function at the community level are inherently difficult to characterize due to multiple, and subtle dynamics. This is further complicated by seasonal and environmental changes.

Biolog plates were used to estimate potential substrate utilization of the bacterial communities. The method represents more specialized functions of the community, which may infer physiological changes to the functional capacity of the communities. Biolog plates have found use for this purpose (e.g., Sala et al., 2006; Comte and Del Giorgio, 2009; Berggren and Giorgio, 2015), despite methodological concerns, e.g., culturing and select substrates. The capacity of substrate utilization in the communities changed over the year, with some substrates being utilized on all occasions, and others only occasionally. Substrates utilized on all occasions suggest that the given substrate is utilized by a broad assembly of bacteria within the community and its utilization can be considered as a core function for the particular community. On the other hand, substrates with periodic utilization are of more specialized character which may depend on the presence of specific populations. While the variance in substrate utilization was significantly correlated to the month of the year, no correlation was found between the composition of the total or active bacterioplankton communities and the patterns of substrate capacity inferred by the Biolog assay, similar to an observed dissimilarity between the substrate capacity and composition of boreal freshwater bacterioplankton communities (Ruiz-González et al., 2015a). This may again be tied to a functional redundancy in the utilization of some substrates, while it also suggests that the potential substrate capacity of a community is not necessarily tied to major community dynamics, but rather, to more subtle changes among non-prominent populations.

In the present study, we identified specific bacterioplankton populations from the present major phyla, e.g., *Cyanobacteria, Bacteroidetes, Actinobacteria, Alpha-*, and *Gammaproteobacteria*, as potential important contributors to community functions, in two contrasting coastal systems. Notably, the correlation between a *Synechococcus* population and protease activity shows how a single population may drive a community function. In combination with the overall prominence of DOM-related variables in our models, it suggests that the bacterioplankton respond dynamically to their contemporary substrate landscape, with a particular importance of nitrogenous DOM and the supply and quality of DOM. Our work exemplifies that high taxonomic resolution may provide unique insights into the populations defining bacterioplankton functions, and that for some taxa rRNA analyses is a necessary descriptor when attempting to correlate functions with communities.

## Author Contributions

ST, LR, SM, JH, MB-T, MM, and HK-L conceived the study. ST, MB-T, MM, and HK-L carried out the field work and the experimental measurements. ST, HS, CS, and LR performed the analyses and interpretations. ST and LR wrote the paper including comments and revisions from all co-authors. All authors have read and approved the submitted version.

## Conflict of Interest Statement

The authors declare that the research was conducted in the absence of any commercial or financial relationships that could be construed as a potential conflict of interest.

## Footnotes

1 http://blast.ncbi.nlm.nih.gov/

2 http://www.R-project.org
