# Supplementary material for: Coupling Bacterioplankton Populations and Environment to Community Function in Coastal Temperate Waters
Source: Front Microbiol. 2016 Sep 27;7:1533. doi: 10.3389/fmicb.2016.01533 (PMC5037133; doi:10.3389/fmicb.2016.01533)
Supplement: Supplementary file 1 [file Table_1.PDF]

Supplementary Material

Coupling bacterioplankton populations and environment to community function in coastal temperate waters

Sachia J. Traving, Mikkel Bentzon-Tilia, Helle Knudsen-Leerbeck, Mustafa Mantikci, Jørgen L. S. Hansen, Colin A. Stedmon, Helle Sørensen, Stiig Markager and Lasse Riemann*

*** Correspondence:** Lasse Riemann: lriemann@bio.ku.dk

1 Supplementary Figures and Tables

1.1 Supplementary Tables

Table S1. List of the measured community functions and environmental parameters, included in the LASSO analyses. The full OTU tables of the 16S ribosomal RNA gene (rDNA) and rRNA were included in the LASSO analyses as community variables. Community functions (response variables) represent bacterial functions related to extracellular enzyme activity and bacterial growth. The predictor variables in the LASSO models consisted of bacterial populations from the present (rDNA) and active (rRNA) communities, and environmental parameters.

**Name**	**Description**	**Type**
Lipase	extracellular enzyme	community function
α-glucosidase	extracellular enzyme	community function
β-glucosidase	extracellular enzyme	community function
chitobiase	extracellular enzyme	community function
Protease	extracellular enzyme	community function
BA	bacterial abundance	community function
BP	bacterial production	community function
BGE	bacterial growth efficiency	community function
peak.A	fluorescent component	environmental variable (Coble, 1996)
peak.C	fluorescent component	environmental variable (Coble, 1996)
peak.M	fluorescent component	environmental variable (Coble, 1996)
peak.T	fluorescent component	environmental variable (Coble, 1996)
Fl.Index	ratio em.450/500, ex.370 nm	environmental variable (McKnight et al., 2001)
a_{255}	absorbance at 255 nm	environmental variable
a_{300}	absorbance at 300 nm	environmental variable
a_{351}	absorbance at 351 nm	environmental variable
a_{375}	absorbance at 375 nm	environmental variable

$S_{275-295}$	slope between 275-295 nm	environmental variable
$S_{300-450}$	slope between 300-450 nm	environmental variable
$S_{300-650}$	slope between 300-650 nm	environmental variable
C1	PARAFAC component	environmental variable
C2	PARAFAC component	environmental variable
C3	PARAFAC component	environmental variable
C4	PARAFAC component	environmental variable
Temperature	°C	environmental variable
Salinity	PSU	environmental variable
pH		environmental variable
Chl *a*	Chlorophyll *a*	environmental variable
Phot. Max	photosynthesis maximum	environmental variable
TN	total nitrogen	environmental variable
TDN	total dissolved nitrogen	environmental variable
PON	particulate organic nitrogen	environmental variable
DIN	dissolved inorganic nitrogen	environmental variable
NO_2^-	nitrite	environmental variable
NO_3^-	nitrate	environmental variable
NH_4^+	ammonium	environmental variable
TP	total phosphorus	environmental variable
PO_4^{-3}	phosphorus	environmental variable
Si	silicate	environmental variable
TOC	total organic carbon	environmental variable
DOC	dissolved organic carbon	environmental variable
BDOC%	bioavailable DOC in % of DOC	environmental variable
POC	particulate organic carbon	environmental variable

Table S2. LASSO models for each community function which contains one or more variables with correlations passing the criteria of occurring in both the Roskilde Fjord (RF) and Great Belt (GB) dataset and with identical sign of the correlation. The RF/GB corr and R^2 are Pearson's correlations between the community function and variables representing environmental parameters or bacterial populations (operational taxonomic units, OTUs). RMSPE = root mean squared prediction error, and please note that this maintains the unit of the function variable. Variables in bold indicate R^2 > 0.4. Abbreviations: ^{D} = 16S ribosomal DNA, ^{R} = 16S ribosomal RNA, Fl.Index =Fluorescence index, Phot.max = photosynthesis maximum, BP = bacterial production, BA = bacterial abundance, BGE = bacterial growth efficiency.

**Model(function)**	**Variable**	**RFcorr**	**GBcorr**	**R^2**	**RMSPE**
**Lipase**	OTU_24^D	-0.335	-0.189	0.325	3.378
	OTU_27^D	0.116	0.035	0.270	3.513
	a_{255}	-0.078	-0.464	0.272	3.537
**α-glucosidase**	OTU_24^D	-0.126	-0.303	0.057	3.104
	OTU_101^D	0.534	0.170	0.108	3.020
	OTU_17^R	0.408	0.364	0.157	2.935
**β-glucosidase**	OTU_35^R	-0.539	-0.064	0.030	9.048
	$S_{300-450}$	0.324	0.523	0.251	8.233
**Chitobiase**	$S_{300-450}$	0.307	0.560	0.325	6.650
**Protease**	**OTU_55^R**	**0.234**	**0.639**	**0.511**	**83.826**
	**Fl.Index**	**-0.609**	**-0.304**	**0.480**	**88.194**
	**a_{255}**	**0.041**	**0.446**	**0.430**	**92.328**
	**NO_2^-**	**-0.235**	**-0.099**	**0.423**	**92.889**
**BP**	OTU_6^R	-0.457	-0.605	0.303	4.361
	$S_{275-295}$	-0.331	-0.199	0.079	5.268
	$S_{300-450}$	0.353	0.166	0.045	5.365
	Chl *a*	0.397	0.224	0.116	5.160
	Si	-0.426	-0.450	0.158	5.037
**BA**	**$S_{275-295}$**	**-0.232**	**-0.013**	**0.598**	**1535702**
	**TDN**	**0.353**	**0.135**	**0.594**	**1544442**
**BGE**	OTU_24^D	-0.183	-0.079	0.061	11.433
	OTU_35^R	-0.126	-0.135	0.057	11.452

1.2 Supplementary Figures

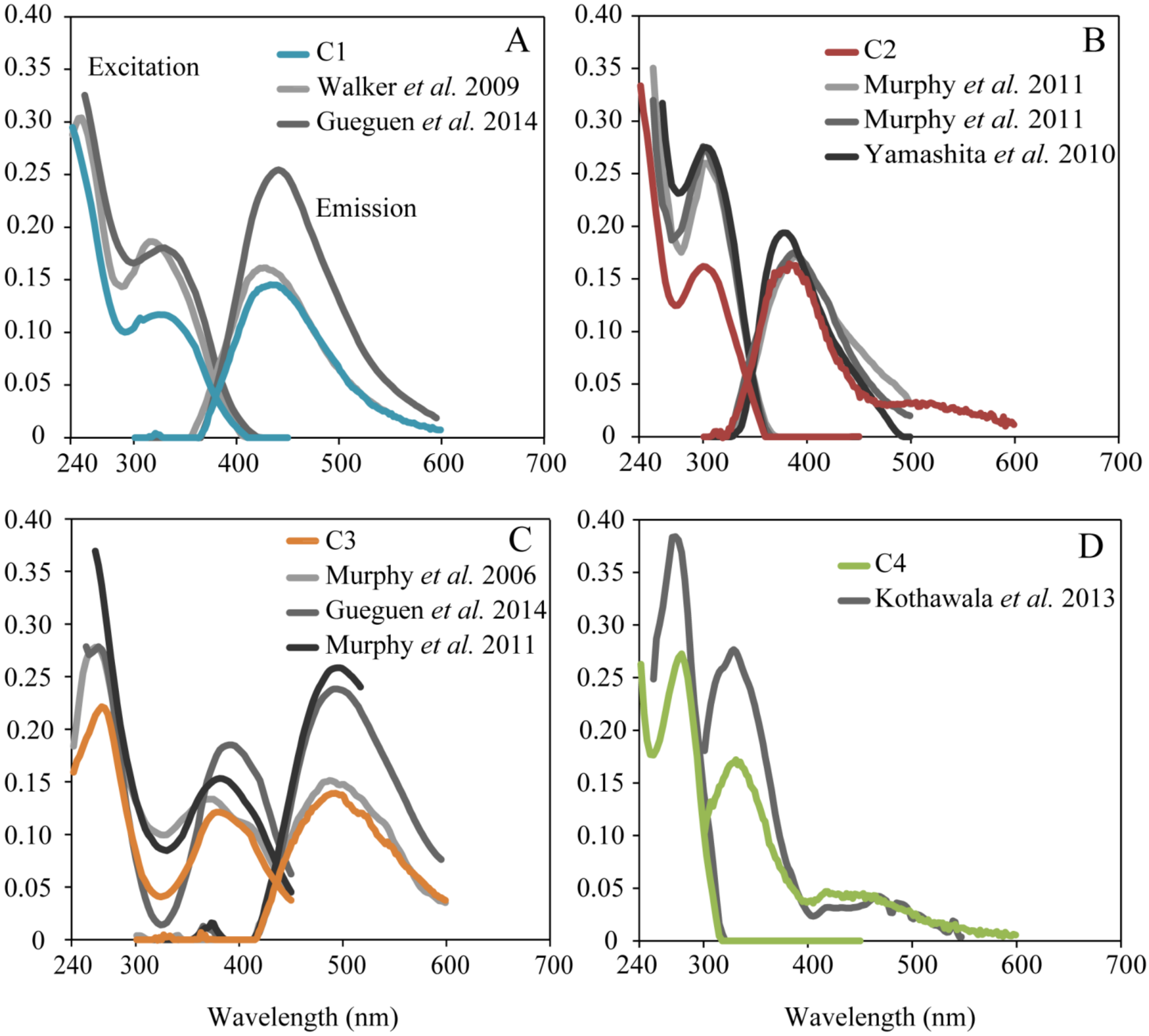

Figure S1. Excitation and emission spectra of the four colored dissolved organic matter (CDOM) components identified in the PARAFAC analysis C1-4. The spectral properties of the four components were analyzed using the OpenFluor database (Murphy et al., 2014) identifying similar components which link C1 to a terrestrial humic-like component (Yamashita et al., 2010, Walker et al., 2009, Guéguen et al., 2014), C2 to a microbial humic-like component (Yamashita et al., 2010, Murphy et al., 2011), C3 to a terrestrial humic-like component (Murphy et al., 2008, Murphy et al., 2006, Guéguen et al., 2014), and C4 to a protein-like component (Kothawala et al., 2014).

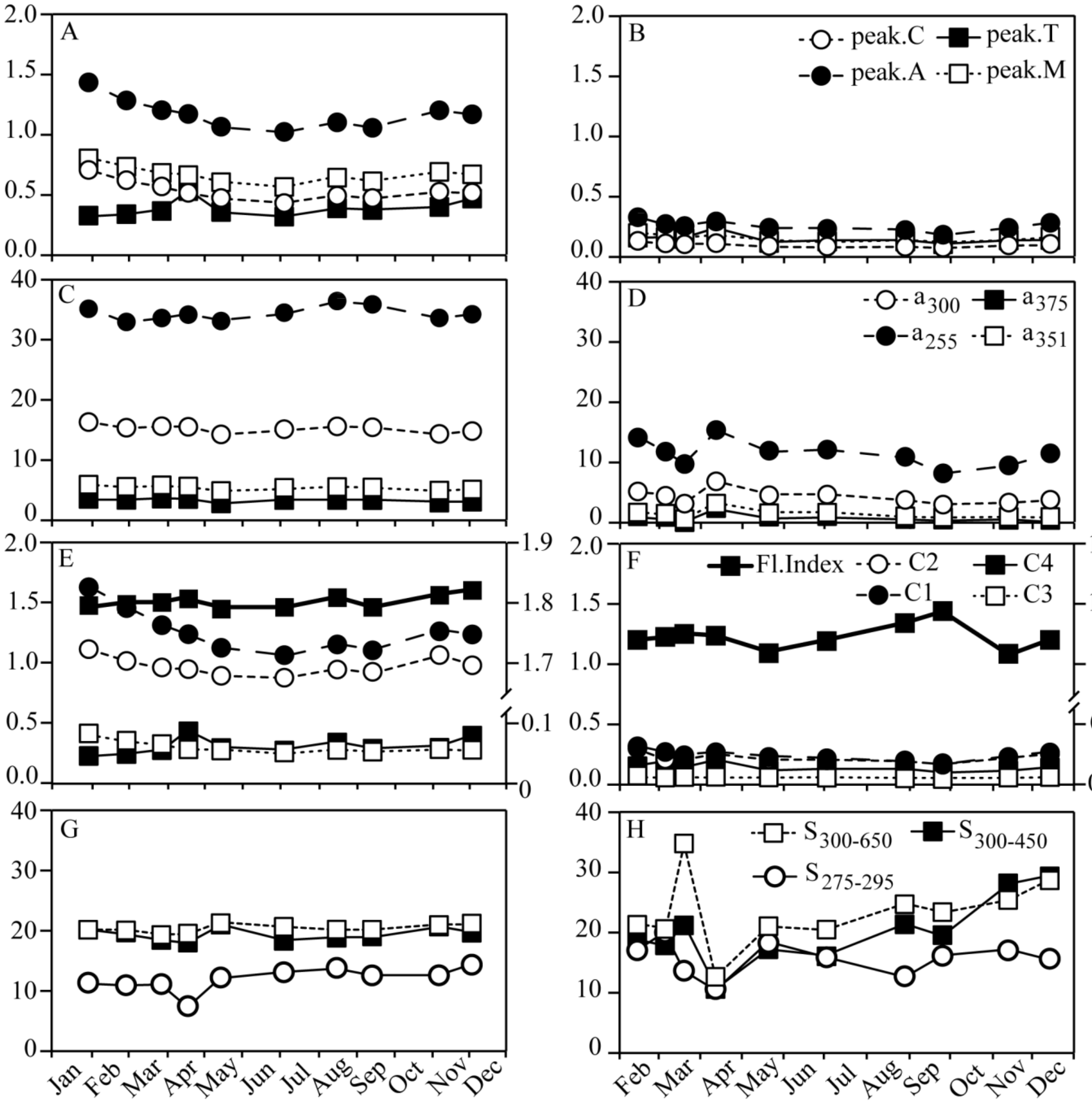

Figure S2. Colored dissolved organic matter (CDOM) parameters for Roskilde Fjord (left side panels) and the Great Belt (right side panels) over the year. Previously identified excitation-emission peaks as described by Coble (1996), in Roskilde Fjord (A) and Great Belt (B). Absorbances at different wavelengths are shown in (C) and (D). PARAFAC components identified in this study (E) and (F) on the left side y-axes and the Fluorescence index (Fl. Index) on right side y-axes. The slope in absorption range (G) and (H) absorption spectral slope ratios and the fluorescence index as defined by McKnight et al. (2001).

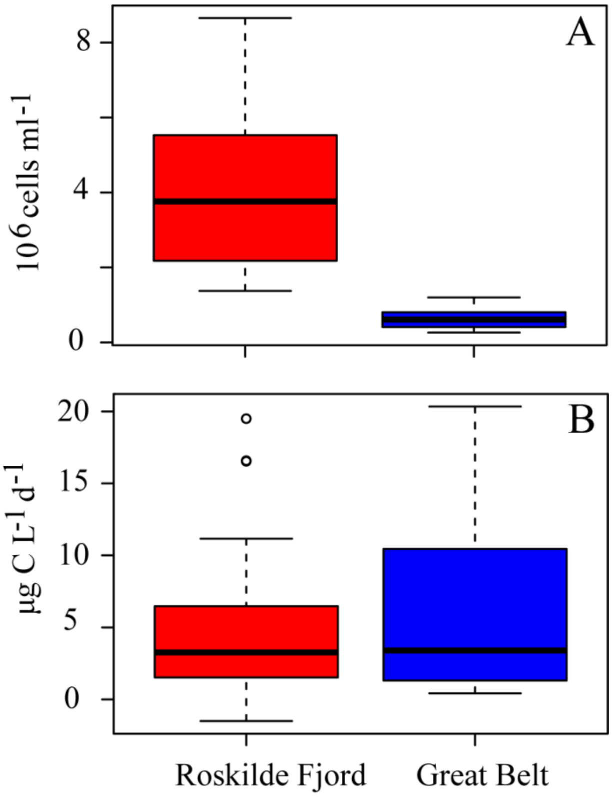

Fig. S3. Box and Whisker plots indicating the median with the lower 25th and upper 75th percentile, of bacterial abundance (A) and bacterial production (B) over the year in Roskilde Fjord (red) and Great Belt (blue).

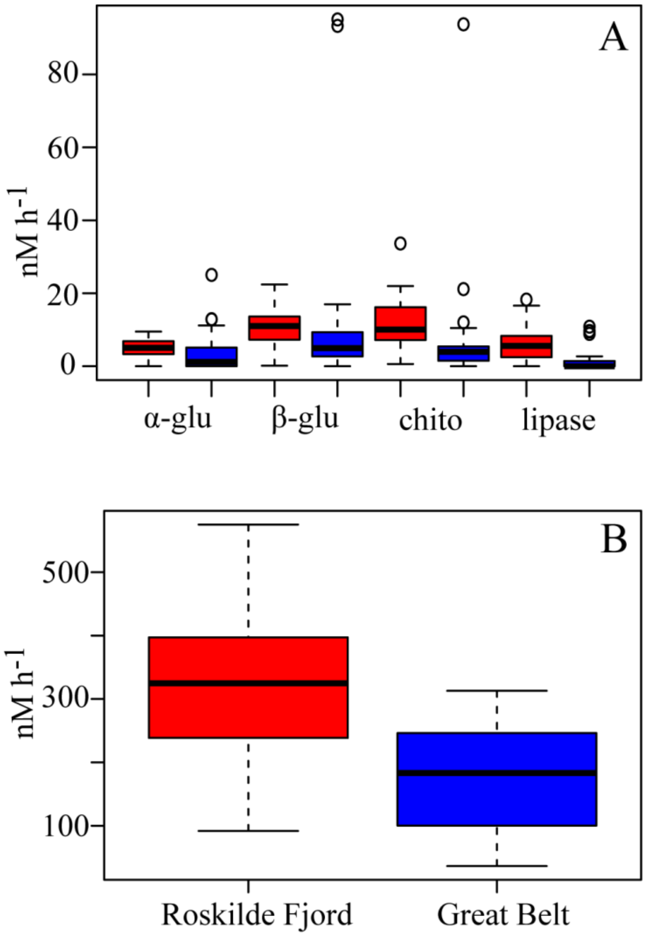

Fig. S4. Box and Whisker plots of extracellular enzyme activities over the year in Roskilde Fjord (red) and Great Belt (blue). The activity of α-glucosidase (α-glu), β-glucosidase (β-glu), chitobiase (chito) and lipase (A) and protease (B).

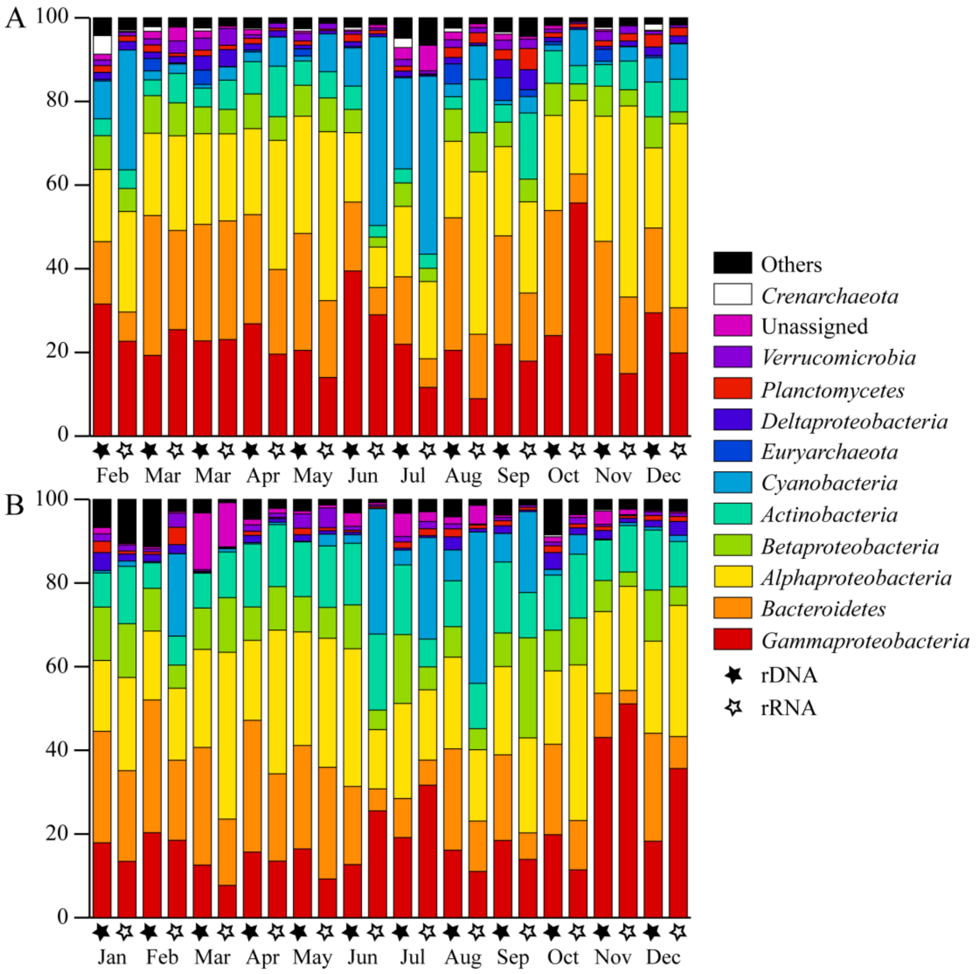

Figure S5. Stacked bars of compositions at the phylum level over time, in the total community (rDNA) and active community (rRNA) in Roskilde Fjord (A) and Great Belt (B). The phylum *Proteobacteria* is resolved to class level. “Unassigned” contains OTUs where the taxonomy was not resolved beyond *Bacteria* in the Greengenes v. 13.8 database. “Others” contain “Proteobacteria_others”, *Epsilonproteobacteria*, *Firmicutes*, *Tenericutes*, *Acidobacteria*, *Chloroflexi*, *Chlorobi*, *Gemmatimonadetes*, *Fusobacteria*, *Chlamydiae*, SAR406, *Lentisphaerae*, *Nitrospirae*, WS3, *Caldithrix*, TM6, *Fibrobacteres*, ZB3, [Thermi], WPS-2, TA18, *Spirochaetes*, [Caldithrix], H-178, [Parvarchaeota], GN02, OD1, OP3, PAUC34f, WS2, OP8, WWE1, *Armatimonadetes*, SR1, BRC1, *Deferribacteres*, *Elusimicrobia*, GN04, LD1, NKB19, OP9, TM7 and *Zetaproteobacteria*.

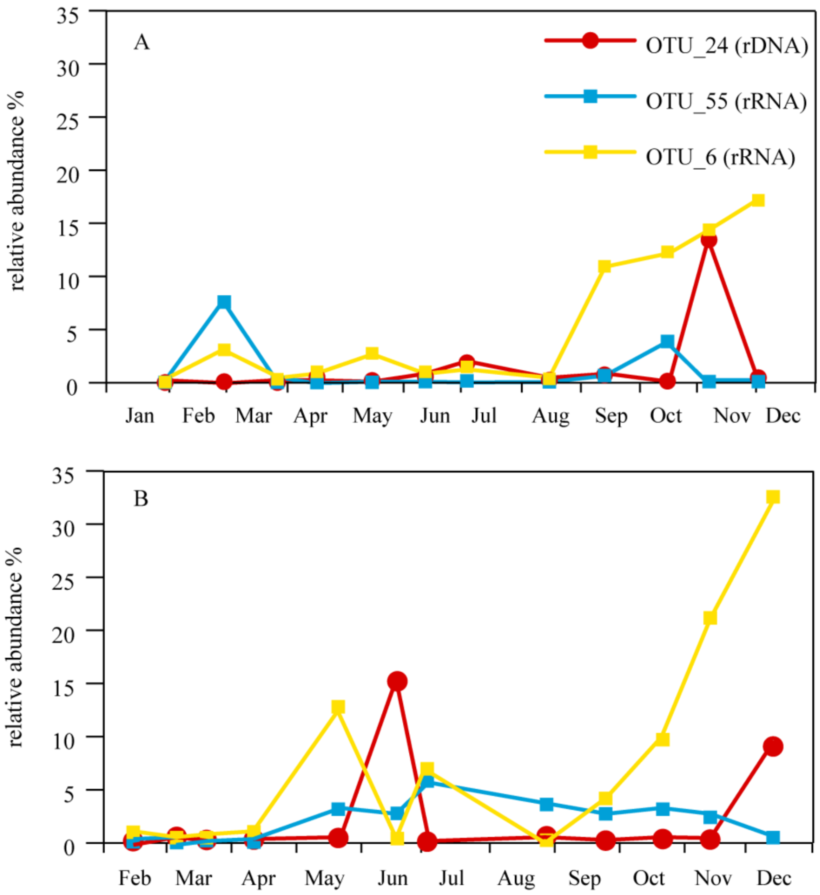

Figure S6. The relative abundance, as proportion of total number of reads in a sample, using the subsampled communities with 16000 reads/sample in depth. The relative abundance of OTU_24 - a population of *Pseudoalteromonas* (*Gammaproteobacteria*) in the present (total) communities, and the relative abundance in the active communities of OTU_55 - a population of *Synechococcus* (*Cyanobacteria*) and OTU_6 - a population of *Caulobacteraceae* (*Alphaproteobacteria*), in Roskilde Fjord (A) and Great Belt (B) over the year.

2 References

COBLE, P. G. 1996. Characterization of marine and terrestrial DOM in seawater using excitation-emission matrix spectroscopy. *Marine Chemistry,* 51**,** 325-346.

GUÉGUEN, C., CUSS, C. W., CASSELS, C. J. & CARMACK, E. C. 2014. Absorption and fluorescence of dissolved organic matter in the waters of the Canadian Arctic Archipelago, Baffin Bay, and the Labrador Sea. *Journal of Geophysical Research: Oceans,* 119**,** 2034-2047.

KOTHAWALA, D. N., STEDMON, C. A., MÜLLER, R. A., WEYHENMEYER, G. A., KÖHLER, S. J. & TRANVIK, L. J. 2014. Controls of dissolved organic matter quality: evidence from a large-scale boreal lake survey. *Global change biology,* 20**,** 1101-1114.

MCKNIGHT, D. M., BOYER, E. W., WESTERHOFF, P. K., DORAN, P. T., KULBE, T. & ANDERSEN, D. T. 2001. Spectrofluorometric characterization of dissolved organic matter for indication of precursor organic material and aromaticity. *Limnology and Oceanography,* 46**,** 38-48.

MURPHY, K. R., HAMBLY, A., SINGH, S., HENDERSON, R. K., BAKER, A., STUETZ, R. & KHAN, S. J. 2011. Organic matter fluorescence in municipal water recycling schemes: toward a unified PARAFAC model. *Environmental science & technology,* 45**,** 2909-2916.

MURPHY, K. R., RUIZ, G. M., DUNSMUIR, W. T. & WAITE, T. D. 2006. Optimized parameters for fluorescence-based verification of ballast water exchange by ships. *Environmental science & technology,* 40**,** 2357-2362.

MURPHY, K. R., STEDMON, C. A., WAITE, T. D. & RUIZ, G. M. 2008. Distinguishing between terrestrial and autochthonous organic matter sources in marine environments using fluorescence spectroscopy. *Marine Chemistry,* 108**,** 40-58.

MURPHY, K. R., STEDMON, C. A., WENIG, P. & BRO, R. 2014. OpenFluor–an online spectral library of auto-fluorescence by organic compounds in the environment. *Analytical Methods,* 6**,** 658-661.

WALKER, S. A., AMON, R. M., STEDMON, C., DUAN, S. & LOUCHOUARN, P. 2009. The use of PARAFAC modeling to trace terrestrial dissolved organic matter and fingerprint water masses in coastal Canadian Arctic surface waters. *Journal of Geophysical Research: Biogeosciences,* 114.

YAMASHITA, Y., SCINTO, L. J., MAIE, N. & JAFFÉ, R. 2010. Dissolved organic matter characteristics across a subtropical wetland’s landscape: application of optical properties in the assessment of environmental dynamics. *Ecosystems,* 13**,** 1006-1019.
